# AAZTA-Derived Chelators for the Design of Innovative Radiopharmaceuticals with Theranostic Applications

**DOI:** 10.3390/ph15020234

**Published:** 2022-02-16

**Authors:** Cyril Fersing, Nicolas Masurier, Léa Rubira, Emmanuel Deshayes, Vincent Lisowski

**Affiliations:** 1 Nuclear Medicine Department, Institut Régional du Cancer de Montpellier (ICM), University Montpellier, 34298 Montpellier, France; lea.rubira@icm.unicancer.fr (L.R.); emmanuel.deshayes@icm.unicancer.fr (E.D.); 2 IBMM, University Montpellier, CNRS, ENSCM, 34293 Montpellier, France; nicolas.masurier@umontpellier.fr (N.M.); vincent.lisowski@umontpellier.fr (V.L.); 3 Institut de Recherche en Cancérologie de Montpellier (IRCM), INSERM U1194, Institut Régional du Cancer de Montpellier (ICM), University of Montpellier, 34298 Montpellier, France; 4 Department of Pharmacy, Lapeyronie Hospital, CHU Montpellier, 191 Av. du Doyen Gaston Giraud, 34295 Montpellier, France

**Keywords:** AAZTA, bifunctional chelator, mesocyclic, radiopharmaceuticals, theranostics, nuclear medicine

## Abstract

With the development of ^68^Ga and ^177^Lu radiochemistry, theranostic approaches in modern nuclear medicine enabling patient-centered personalized medicine applications have been growing in the last decade. In conjunction with the search for new relevant molecular targets, the design of innovative chelating agents to easily form stable complexes with various radiometals for theranostic applications has gained evident momentum. Initially conceived for magnetic resonance imaging applications, the chelating agent AAZTA features a mesocyclic seven-membered diazepane ring, conferring some of the properties of both acyclic and macrocyclic chelating agents. Described in the early 2000s, AAZTA and its derivatives exhibited interesting properties once complexed with metals and radiometals, combining a fast kinetic of formation with a slow kinetic of dissociation. Importantly, the extremely short coordination reaction times allowed by AAZTA derivatives were particularly suitable for short half-life radioelements (i.e., ^68^Ga). In view of these particular characteristics, the scope of this review is to provide a survey on the design, synthesis, and applications in the nuclear medicine/radiopharmacy field of AAZTA-derived chelators.

## 1. Introduction

As an emerging approach in modern medicine, “theranostics” (also called “theragnostics”) consists of the combination of diagnostic and therapeutic tools to achieve personalized patient care [1]. Various scientific disciplines, especially in the field of nanotechnology [2] and biomaterials [3], can be involved in theranostic approaches. Nuclear medicine is particularly suited to this concept, easily combining molecular imaging and targeted radionuclide therapy [4]. Historically, nuclear medicine is deeply connected to theranostics, being used for decades for the management of benign and malignant thyroid diseases with radioiodine isotopes [5,6]. In recent years, theranostic in nuclear medicine benefited from the rise of ^68^Ga and ^177^Lu radiochemistry, allowing easy radiolabeling of the same vector molecule by either a photon-emitting (i.e., ^68^Ga for PET imaging) or a particle-emitting (i.e., ^177^Lu, beta-emitter for therapy) radioelement. This approach is all the more convenient since the radioelements used are metals, which can be readily complexed by a vector molecule functionalized by a chelating group. The “theranostic pair” ^68^Ga/^177^Lu is an illustrative example, successfully applied to the management of several malignancies, in particular neuroendocrine tumors (NETs) [7] and prostate cancer [8,9]. New theranostic pairs have recently become increasingly popular, especially those involving scandium-44. This latter positron-emitting isotope showed high potential for TEP imaging and can be paired with either ^177^Lu or its beta-emitter isotope ^47^Sc for therapeutic applications [10]. Radiolabeling with radiometals requires optimal coordination chemistry conditions, including the use of chelating groups best suited to the theranostic couples used in nuclear medicine. The complexation reaction step can be industrialized for radioelements with a long half-life (i.e., ^177^Lu), whereas radiopharmaceuticals containing short half-life radioelements (i.e., ^68^Ga) have to be prepared extemporaneously, in the radiopharmacy laboratory, under reaction conditions that should be as simple and robust as possible. To date, the chelators of choice for the design of radiopharmaceuticals are either macrocycles such as DOTA (used in DOTATOC, DOTATATE, or PSMA-617) or acyclic groups such as DTPA (used in pentetreotide). However, many new chelating agents with more sophisticated structures and enhanced properties have been developed in recent years [11]. Among these original derivatives, AAZTA (6-amino-6-methylperhydro-1,4-diazepinetetraacetic acid) is a heptadentate chelator formed by a medium-sized, seven-membered 1,4-diazepine ring and an iminodiacetic exocyclic group. This particular structure makes it part of the mesocyclic chelating agents class. The first of these chelators, AAZTA, was initially designed to form gadolinium complexes of particular interest in MRI imaging. In addition to forming a stable complex, Gd-AAZTA could bind with two water molecules in its coordination sphere (hydration number *q* = 2), reaching high relaxivities. Other chelators inspired by AAZTA were then developed and displayed promising coordination properties for some radiometals such as gallium-68 or scandium-44. Although AAZTA and its derivatives have been thoroughly studied for their possible applications in magnetic resonance imaging (MRI), the use of these chelators for radiochemical applications has only become widespread in recent years. The aim of this article is therefore to present an overview of the main information related to AAZTA-derived chelators and their applications in radiopharmacy and nuclear medicine. Particular emphasis will be placed on synthetic routes to obtain these chelators. Key parameters such as the thermodynamic stability (determining the extent to which the complex will be formed or be converted into another complex at the point of equilibrium) and the kinetic stability (describing the reaction rate and the reactivity of a metal complex in solution) of the resulting metal complexes will be considered. Derivatization of AAZTA chelators into bifunctional agents, as well as the design and evaluation of original radiopharmaceuticals candidates containing this type of chemical moiety, will finally be covered.

## 2. Design, Synthesis, and Kinetic Properties

### 2.1. Original AAZTA Derivatives

**Scheme 1:**
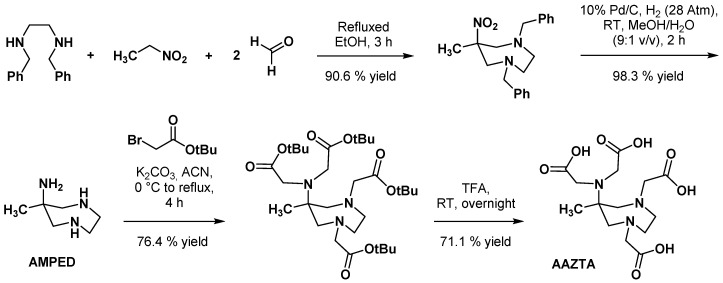
Original synthesis sequence of AAZTA described by Aime et al. [12].

The first synthesis of AAZTA was reported by Aime et al. in 2004, presenting this chelator as a promising MRI contrast agent candidate [12]. The first step of its synthesis sequence (Scheme 1) consisted of the formation of the diazepane ring via a nitro-Mannich reaction. Heating a mixture of *N,N′*-dibenzylethylenediamine diacetate, nitroethane, and formaldehyde in ethanol allowed the formation of 1,4-dibenzyl-6-methyl-6-nitroperhydro-1,4-diazepine in excellent yields. Subsequent reduction of the nitro group and simultaneous bis-*N*-debenzylation catalyzed by palladium on carbon in a methanol/water mixture under hydrogen atmosphere led to 6-amino-6-methylperhydro-1,4-diazepine (AMPED), a strongly basic triamine whose nitrogen atoms can be functionalized. Complete alkylation of both the primary amine and the two secondary amines using *tert*-butylbromoacetate with potassium carbonate in acetonitrile formed the final protected derivative, subsequently treated by trifluoroacetic acid to obtain the AAZTA chelator with a 48% overall yield. In addition to being simple and straightforward, this synthesis has the advantage of using readily available and cheap reactants.

Evaluated for its potentiometric and relaxometric properties, the [Gd-AAZTA]^−^ complex showed a stability constant (log*K*) of 19.26, slightly smaller than [Gd-DTPA]^2−^. However, this complex displayed no transmetallation effects in the presence of Zn, Mn, or Ca, an exchange rate of the two coordinated water molecules significantly higher than [Gd-DTPA]^2−^ and [Gd-DOTA]^−^ (90 ns vs. 300 ns and 240 ns, respectively) and a high kinetic inertness (unchanged relaxivity at 20 MHz and 298 K for a pH range from 2 to 11). Coordination behavior of AAZTA with other lanthanoids was very comparable, with a monotonous increase in the complexes’ stability from La^3+^ to Lu^3+^ but with log*K* values slightly lower than those of the analogous DTPA complexes [13]. Complementary studies confirmed the excellent kinetic inertness of the [Gd-AAZTA]^−^ complex near physiological conditions (t_1/2_ = 4337 h vs. 147 h for [Gd-DTPA]^2−^). The [Mn-AAZTA]^2−^ complex was also studied with a view to its application as an MRI probe [14]. For this smaller transition metal, a much higher kinetic inertness was observed for complexes with rigidified analogues of EDTA than with AAZTA. Concerning group 13 metals, stability constants of the complexes formed with the AAZTA ligand were also generally lower than the corresponding DTPA complexes, especially with small atoms such as Ga^3+^ (log*K*_Ga-AAZTA_ = 22.18 vs. log*K*_Ga-DTPA_ = 24.3) [15]. The same applies to [Cu-AAZTA] (log*K*_Cu-AAZTA_ = 22.27 vs. log*K*_Cu-DTPA_ = 23.4), probably due to an unfavorable size match between the metal and the rigidified coordination cage of AAZTA, in a manner comparable to [Mn-AAZTA]^2−^. Interestingly, the titration data for [Ga-AAZTA]^−^ obtained at pH > 3 indicated the occurrence of a base-consuming process related to the competition between an OH^−^ ion and AAZTA for Ga^3+^, forming the [Ga-AAZTA-OH]^2−^ complex. This latter was the predominant species in the pH range 6–10, formed within seconds in experimental conditions. It contrasts with the slow formation kinetics of DOTA chelators, requiring heating and long reaction times. The [Ga-AAZTA-OH]^2−^ complex displayed high kinetic inertness towards dissociation (t_1/2_ = 24 h at pH 7.5 and 25 °C in 0.025 M NaHCO_3_ and 0.15 M NaCl), transmetallation (t_1/2_ = 21 h with copper citrate), and transferrin-mediated demetallation (t_1/2_ = 23 h). These promising properties were therefore in favor of AAZTA chelators in the design of ^68^Ga-based radiopharmaceutical candidates. This possible application could also be extended to ^44^Sc-based PET imaging agents [16]. Indeed, the study of the [Sc-AAZTA]^−^ complex highlighted a conditional stability about six times higher than [Sc-DOTA]^−^ despite a lower stability constant (log*K*_Sc-AAZTA_ = 27.69 vs. log*K*_Sc-DOTA_ = 30.79) [17]. Radiolabeling conditions with ^44^Sc were less demanding for the AAZTA chelator, which required a lower concentration than DOTA to reach similar or better radiochemical yields (RCY). It also made it possible to operate in a broader pH range (even more so with high chelator concentrations) and at lower temperatures (>95% incorporation of ^44^Sc achieved almost instantaneously at room temperature for [AAZTA] > 1 µM). As for the previously studied metals, the scandium complex with AAZTA was characterized by impressive kinetic stability (extrapolated t_1/2_ = 5.6 years at pH = 7.4) and an in vitro plasma stability over 12 h, similar to [Sc-DOTA]^−^. Overall, these results suggested AAZTA compatibility with Ga, Sc, and Lu and the possibility of a safe in vivo use of potential AAZTA-radiopharmaceuticals, especially for the labelling of heat-sensitive biomolecules such as antibodies. The interest of chelators allowing fast complexation at room temperature is major in the design of new radiopharmaceuticals. The best example is HBED, a phenol-containing aliphatic chelator able to form stable complexes with ^68^Ga at room temperature within minutes [18,19] and found in PSMA-11, widely used in clinics for prostate cancer PET imaging [20].

In an attempt to improve several properties of the corresponding complexes, especially thermodynamic stability and kinetic inertness, various modulations of the original AAZTA chelator were undertaken. These modifications consisted essentially of either the replacement of one or more acetic arms or the structural stiffening of the chelator by introducing a cyclic group into the ligand backbone.

### 2.2. AAZTA Modulations to Improve Coordination Behavior

#### 2.2.1. Modifications in the Number and Nature of Coordinating Side Arms

**Scheme 2:**
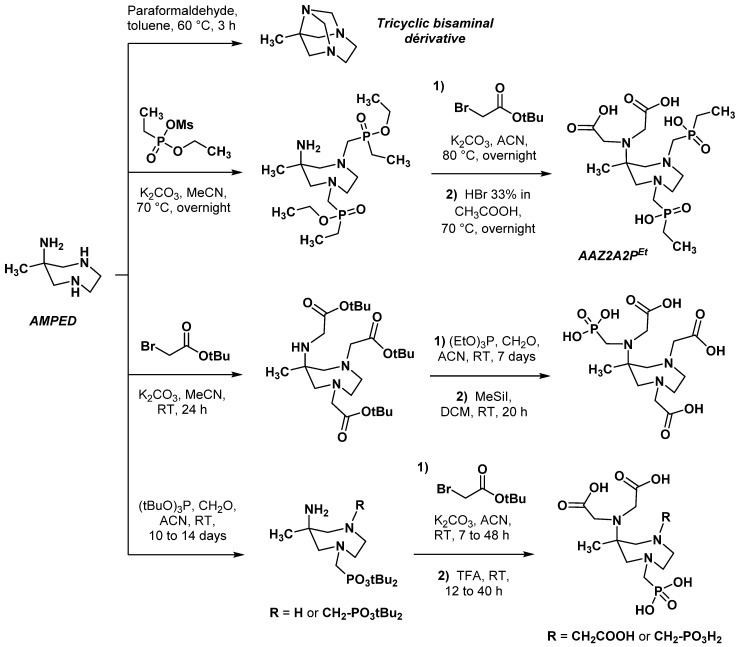
Synthesis sequence of phosphorus-containing AAZTA derivatives described by Ermelindo et al. [21] and Guanci et al. [23]. Ms = methanesulfonate.

Replacement of the two acetic arms in the 1,4 position of AAZTA by bulkier methyleneethylphosphinic groups was reported by Emerlindo et al. in 2013 [21]. Because the Mannich reaction between AMPED, paraformaldehyde, and alkylphosphinate ester quantitatively formed a tricyclic bisaminal derivative in place of the expected product, the phosphinic pendant arms were introduced first, in the form of a methanesulfonic acid ethoxymethylphosphinyl ethyl ester. Alkylation of AMPED with this latter occurred preferentially at endocyclic nitrogen atoms (Scheme 2), most certainly due to both high steric hindrance at the 6-amino position and high nucleophilicity of the secondary amines [13,22]. *N*,*N*-Dialkylation of the 6-amino position with *ter*-butyl bromoacetate was conducted afterward to obtain, after deprotection by hydrobromic acid, the AAZ2A2P^Et^ chelator in 7% overall yields. The [Gd- AAZ2A2P^Et^] complex was characterized by a coordination environment very similar to [Gd-AAZTA], except for its reduced number of coordinated water molecules (*q* = 1, possibly due to the steric hindrance of the phosphinate groups). Most importantly, this work showed the possibility of starting from AMPED as a scaffold for chelating arms’ modulation.

To modulate more finely the number of coordinated water molecules in phosphorus AAZTA derivatives, Guanci et al. synthesized mono- and diphosphonic analogues of AAZTA with a modified arm bore by the exocyclic amine, or one cyclic amine, or both amines of the diazepane ring [23]. Interestingly, the authors did not report the formation of the unwanted tricyclic bisaminal during the Mannich reaction involving AMPED, formaldehyde, and tri*tert*-butyl phosphite (Scheme 2).

These chelators, only studied for their relaxometric properties, displayed once again a reduction in the available space for directly coordinated water molecules (*q* = 0.4 to 1). As this has not been performed yet, it would be interesting to evaluate these mesocyclic ligands as chelators of radiometals such as ^68^Ga by analogy with the phosphorus-containing macrocyclic ligands TRAP [24,25] or NOPO [26].

**Figure 1:**
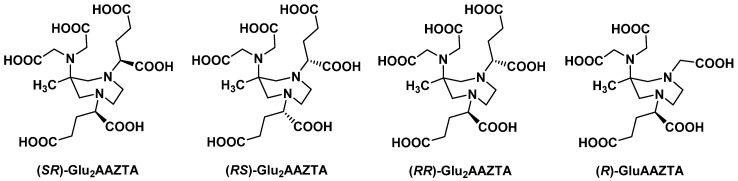
Examples of mono- and di-glutarate analogues of AAZTA synthesized by Elemento et al. [22].

AAZTA derivatives bearing a glutar-2-yl group at one or both endocyclic nitrogen atoms were synthesized by Elemento et al. [22]. Selective alkylation relied on the preferential reactivity of the secondary amines and gave rise to several diastereoisomers (Figure 1). Water exchange rates measured for Gd^III^ complexes of mono- and di-glutarate derivatives were slightly lower than for Gd-AAZTA, with a metal ion hydration number *q* ≈ 2 for each complex. Interestingly, faster exchange dynamics were observed for the (*RS*) derivative compared to its (*RR*) diastereoisomer, suggesting that small modulations on the diazepane ring substituents could increase the steric demand at the metal center. Evaluation of such chelators as radiometal complexing agents would be relevant, especially with regard to the free carboxylate groups on the glutarate arms that could offer the possibility for conjugation reactions.

Guanci et al. reported an AMPED-derived octadentate chelator with three picolinate arms that could form luminescent lanthanide complexes [27]. Its straightforward synthesis consisted of direct mono-*N*-alkylation of both the primary amine and the secondary amines of AMPED with methyl 6-chloromethylpicolinate, followed by deprotection of the carboxylic acid functions. Interestingly, the authors stated that the tetra-alkylated compound could not be isolated, probably because of the steric hindrance generated by the first picolinate arm on the exocyclic amine. Complexes were formed with Eu and Tb, showing the coordination sphere to be fully saturated by the nine coordination sites of the ligand. This excellent compatibility with lanthanide cations could position this type of chelator as a promising complexing agent for lutetium-177 or terbium radioisotopes, in the same way as the acyclic derivative H_4_octapa [28]. Moreover, the fast and facile formation of terbium complexes would be of great value in the design of theranostic tools in nuclear medicine, as radioisotopes of this element form a quadruplet family compatible with SPECT imaging (^155T^b), TEP imaging (^152^Tb), β^−^ therapy (^161^Tb), and α therapy (^149^Tb) [29].

**Figure 2:**
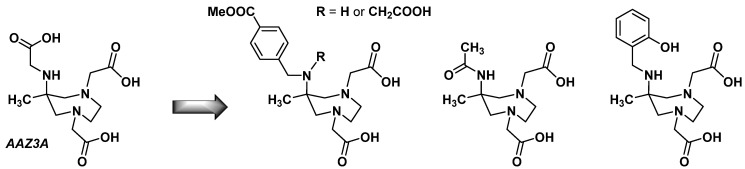
Structure of polydentate mesocyclic ligands described by Martinelli et al. [30].

Other symmetrical or unsymmetrical penta- or hexadentate ligands, characterized by different donor groups and global charges, were synthesized by Martinelli et al. [30]. Most of these derivatives have a secondary exocyclic amine, such as AAZ3A (Figure 2). The modifications proposed on these ligands are interesting leads for both the optimization of their complexing properties (not studied by Martinelli et al.) and for modulations of their physicochemical behavior.

#### 2.2.2. Backbone Rigidification

**Scheme 3:**
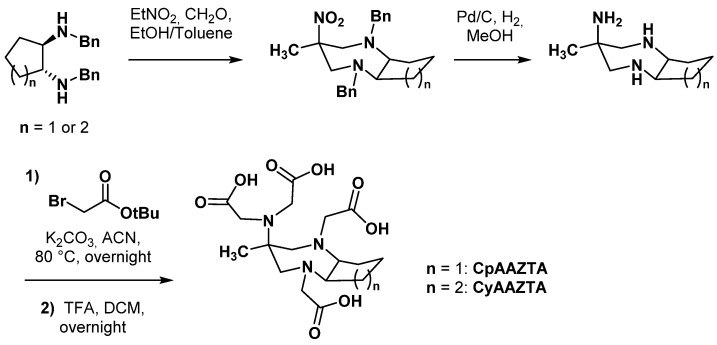
Synthesis of constrained chelators CyAAZTA [33] and CpAAZTA [35].

Structure rigidification by fusing a cyclic unit to the backbone of a chelating agent is a strategy that has already been applied to acyclic ligands such as DTPA. The cyclohexyl ring of CHX-A″-DTPA made the complexing agent stiffer, imposing a degree of preorganization on the metal ion binding site and enhancing its kinetic inertness [31,32]. With the same goal, a cyclohexyl-fused AAZTA chelator (CyAAZTA) was synthesized and studied [33]. CyAAZTA was obtained following a synthesis sequence analogous to AAZTA, starting from *trans*-1,2-dibenzylaminocyclohexane (Scheme 3). The stability constant of [Ga-CyAAZTA]^−^ was slightly lower than [Ga-AAZTA]^−^ (log*K*_Ga-CyAAZTA_ = 21.39 vs. log*K*_Ga-AAZTA_ = 22.18). T_1/2_ values at pH = 6.0 of the predominant species [Ga-CyAAZTA] and [Ga-CyAAZTA-OH]^2−^ were 234 h and 64 h, respectively, probably explained by the constrained structure of CyAAZTA. However, the higher formation of [Ga-CyAAZTA-OH]^2−^ complex at pH > 6 was also shown to alter the kinetic inertness of the complex at physiological pH (t_1/2(Ga-CyAAZTA)_ = 8.5 h vs. t_1/2(Ga-AAZTA)_ = 21 h in transmetallation conditions). An early radiolabeling reaction conditions study showed that a >80% RCY could be reached at room temperature (RT) and pH 3.8 within 15 min, whereas heating at 90 °C during 5 min led to near-quantitative yields. Lanthanide complexes with CyAAZTA were also investigated and displayed stability constants significantly lower than those of the corresponding AAZTA complexes (log*K*_Lu-CyAAZTA_ = 17.67 vs. log*K*_Lu-AAZTA_ = 21.85) [34]. In contrast, a remarkably high kinetic inertness was demonstrated for [Gd-CyAAZTA], but this parameter was not investigated for other lanthanide complexes.

A cyclopentyl-fused analogue of CyAAZTA, called CpAAZTA, was also reported [35]. Its synthesis was comparable in every way with that of CyAAZTA, starting from 1,2-epoxycyclopentane as the precursor of *trans*-1,2-dibenzylaminocyclopentane (Scheme 3). Despite its higher torsional strain, CpAAZTA complexed with Gd^III^ displayed slightly lower kinetic inertness than its CyAAZTA analogue in acidic media.

**Scheme 4:**
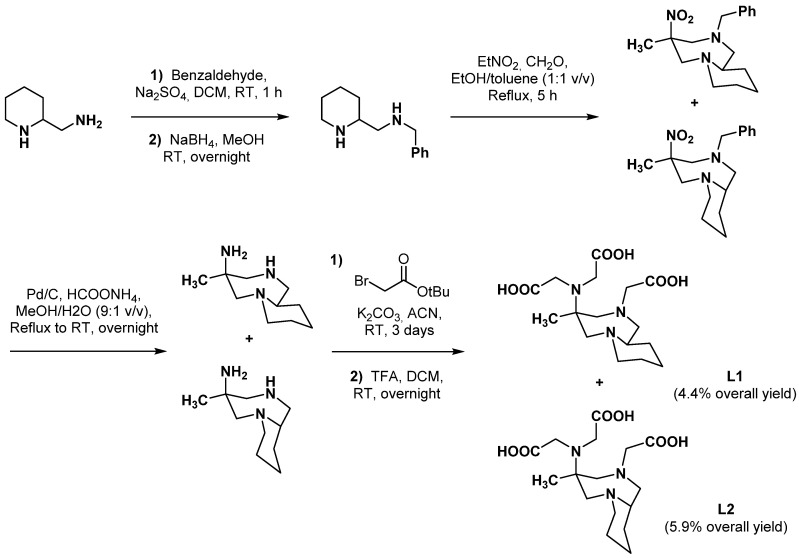
Synthesis of the two PIDAZTA isomers reported by Farkas et al. [36].

The hexadentate ligand PIDAZTA, rigidified by a six-membered ring incorporating one of the coordinating nitrogen atoms, was designed to better fit ^68^Ga [36]. The double nitro-Mannich reaction on 2-(*N*-benzylaminomethyl)piperidine led to two bicyclic isomers (Scheme 4) that were both evaluated for their coordination properties with gallium. Ligand L2 displayed the most promising properties, with a stability constant significantly higher than L1 (log*K*_Ga-L2_ = 21.70 vs. log*K*_Ga-L1_ = 18.77) and similar to [Ga-AAZTA]^−^, [Ga-CyAAZTA]^−^, and [Ga-DATA^m^]. As already described, the [Ga(L)OH]^−^ species was predominantly formed with both isomers in the pH range 6–8, [Ga(L2)OH]^−^ being characterized by the highest cumulative stability constant among all the Ga complexes with AAZTA derivatives. Moreover, [Ga(L2)OH]^−^ showed the highest kinetic inertness among all the Ga complexes with AAZTA-like ligands (t_1/2_ = 295 h at pH 7.4), including under transchelation reaction conditions. However, despite very good radiolabeling properties (RCY = 93.5% after 5 min at pH 7.5 and RT), L2 has not yet been used to design probes for imaging applications.

### 2.3. AAZTA Modulations to Obtain Bifunctional Chelating Agents (BCA)

**Scheme 5:**
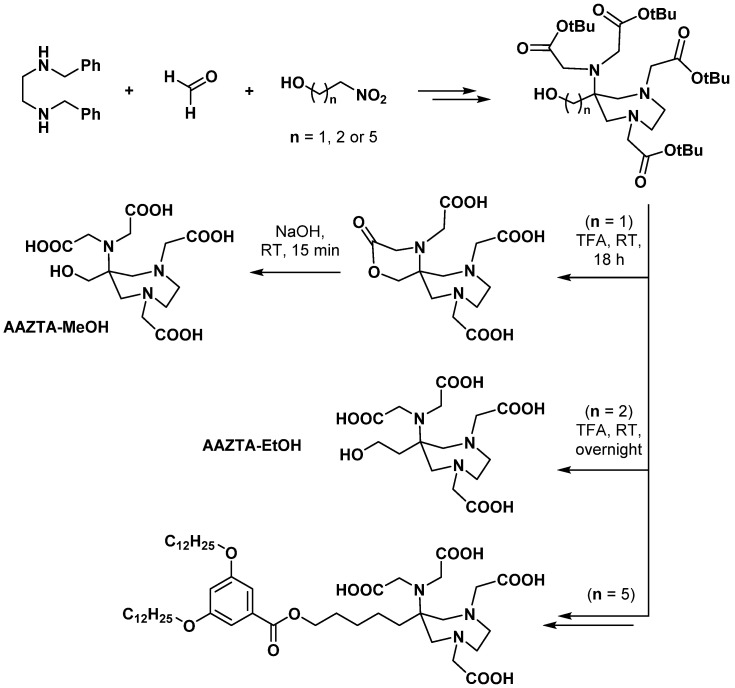
Synthesis of hydroxyalkyl AAZTA derivatives as BCA [39,40].

Various techniques for anchoring a chelating agent to a molecule of interest are described in the literature, each of them requiring the presence of a dedicated reactive group on the chelator to undergo this functionalization [37,38]. In the AAZTA structure, the methyl group on the sp^3^ central carbon atom of the propylene endocyclic moiety is particularly suitable for derivatization as it is exocyclic and not involved in metal ion complexation. Hence, several methyl-modified analogues were designed for their further bioconjugation. Hydroxymethyl AAZTA derivative (AAZTA-MeOH) was described by Sengar et al. and was synthesized following a procedure similar to the original reaction sequence (Scheme 5), nitroethane being replaced with 2-nitroethanol [39]. However, the last deprotection step with trifluoroacetic acid (TFA) led to the formation of a lactone after cyclization between the hydroxyl group with one of the two *tert*-butyl esters located on the exocyclic nitrogen. This unwanted esterification called for an additional basic hydrolysis step prior to the coordination reaction. Interestingly, no such lactonization was observed during the synthesis of the hydroxyethyl AAZTA derivative (AAZTA-EtOH) (Scheme 5), the seven-ring formation being less favorable than the six-ring one. Crystal structures of Gd^III^ and Eu^III^ complexes of AAZTA-MeOH and AAZTA-EtOH showed no direct participation of the hydroxyl side chain in the metal ion chelation, which confirmed this position as an adequate functionalization site. A hydroxypentyl AAZTA derivative was also described by Gianolio et al. and used for the preparation of lipophilic subunits containing two dodecyl chains (Scheme 5) [40]. After complexation with Gd^III^, these molecules were capable of self-assembling in stable particles for cell surface labeling.

**Scheme 6:**
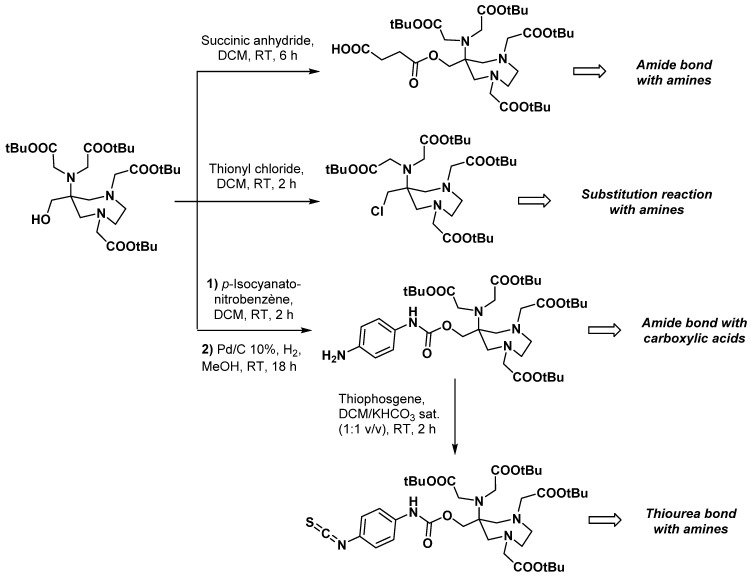
Synthesis of bifunctional chelators derived from AAZTA-MeOH [41,42].

Other reactive groups were considered in the same position as the hydroxyl group of AAZTA-MeOH. Starting from the *tert*-butyl ester analogue of this latter compound, Gugliotta et al. used thionyl chloride to convert the hydroxymethyl group into a chloromethyl moiety (Scheme 6) [41]. The reactivity of this BCA was then demonstrated through its coupling with an amino bile acid derivative. Exploiting the possibility of forming ester bonds with the hydroxymethyl group, the same team synthesized a succinate derivative of AAZTA-MeOH (Scheme 6). Although its reactivity was not exemplified, such a bifunctional agent could be used for conjugation under various conditions (e.g., through amide bond formation).

Gugliotta et al. also described the reaction of AAZTA-MeOH with aromatic isocyanates to form carbamates and thus avoid the risk of intramolecular cyclization with AAZTA-MeOH [42]. Mono- and dimeric BCA containing either an arylamino or an arylisothiocyanato reacting group were synthesized via a convenient two- or three-step procedure, respectively (Scheme 6). Isothiocyanate derivatives, known to readily react with amino-containing molecules, were then linked to a poly(amidoamine) dendrimer generation 1 (PAMAM G1) and a tetrakis(2-aminoethyl)-1,2-ethanediamine molecule to form octameric ligands. Interestingly, the bioconjugation reaction yields seemed to depend on the nature of the molecule to be functionalized (81% yields with PAMAM, 24% yields with the poly-primary amine). Moreover, the use of dichloromethane as a solvent and the overnight reaction time are quite unusual for the functionalization of nuclear imaging vectors, for which an inorganic buffer and slightly shorter reaction times are preferred [43,44,45,46,47].

**Scheme 7:**
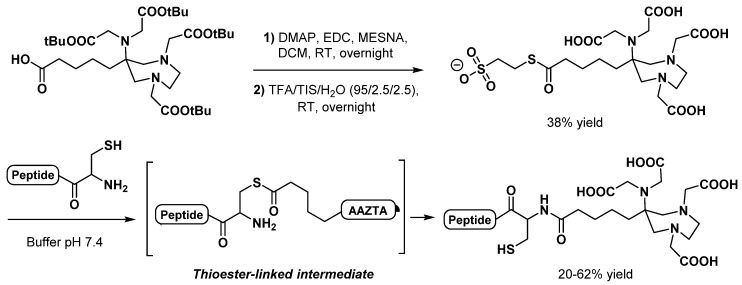
Native chemical ligation mechanism for N-terminal cysteinyl peptides bioconjugation with AAZTA thioester, as reported by Hawala et al. [49].

Another more original linkage method based on the native chemical ligation (NCL) [48] was applied to AAZTA derivatives by Hawala et al. [49]. This technique allowed the formation of an amide bond between a *N*-terminal cysteinyl peptide and a pentanoic acid-AAZTA derivative, preactivated as a sodium 2-mercaptoethanesulfonate thioester (Scheme 7). After the formation of a first thioester bond, subsequent N-S acyl migration led to a free thiol group on the Cys residue, which can be exploited for a second site-specific labeling via the thiol-maleimide chemistry [50]. Ga^III^ complexes of AAZTA-containing AE105 (uPAR agonist) and *cyclo*RGDfK bioconjugates were prepared by mixing equimolar amounts of the functionalized peptide and GaCl_3_ in a 1:1 solution of 0.1 acetate buffer pH 3.8 and acetonitrile, at RT. Both complexes were obtained in high purity (96% and 95%, respectively) without further purification but after 1 h reaction time, which should be shortened to meet standard requirements for ^68^Ga radiolabeling.

Overall, the bioconjugation techniques applied to AAZTA chelators are comparable to those used with other BCA in the construction of radiometal-based radiopharmaceuticals. By analogy, other functional groups such as activated esters (*N*-hydroxysuccinimide-esters, tetrafluorophenyl-esters) for amide couplings, maleimides for thiol couplings, azides or alkynes for Huisgen 1,3-dipolar cycloadditions, and tetrazine or transcyclooctene groups for strain-promoted Diels–Alder reactions could also be considered for the design of AAZTA-based bifunctional chelators [11].

### 2.4. AAZ3A Derivatives

**Scheme 8:**
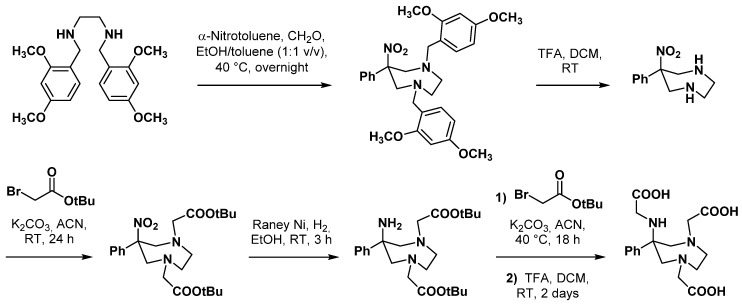
Synthesis of 6-amino-1,4-diazepine triacetate chelating agents [52].

Six-coordinate Ga^III^ complexes are known to reach excellent stability [51], the metal environment forming a more or less distorted octahedral geometry as with Ga-DOTA complexes. To design new hexadentate AAZTA-derived chelators, Parker et al. synthesized several derivatives characterized by an “N3O3” donor set and a single acetate arm on the 6-amino group [52]. These derivatives will be later named DATA chelators after their 6-amino-1,4-diazepine triacetate scaffold. Some of these analogues also incorporated a bulkier 6-phenyl moiety instead of the 6-methyl moiety of AAZTA, so that the 6-amino group is most likely to adopt an axial position that would form a facing-capping array with the three nitrogen atoms. However, this substitution modified the stability of the diazepane ring in such a way that subsequent hydrogenolysis using Pd/C caused ring opening. Acid-labile 2,4-dimethoxybenzyl groups were then used to protect cyclic amines. After deprotection by TFA and alkylation of the two endocyclic nitrogen atoms (Scheme 8), reduction of the nitro group was then achieved with Raney nickel in ethanol at RT. Finally, selective mono-alkylation of the resulting primary amine has been achieved using the appropriate α-bromo ester. Some derivatives also underwent a *N*-methylation reaction of the 6-amino group, using iodomethane in acetonitrile.

**Figure 3:**
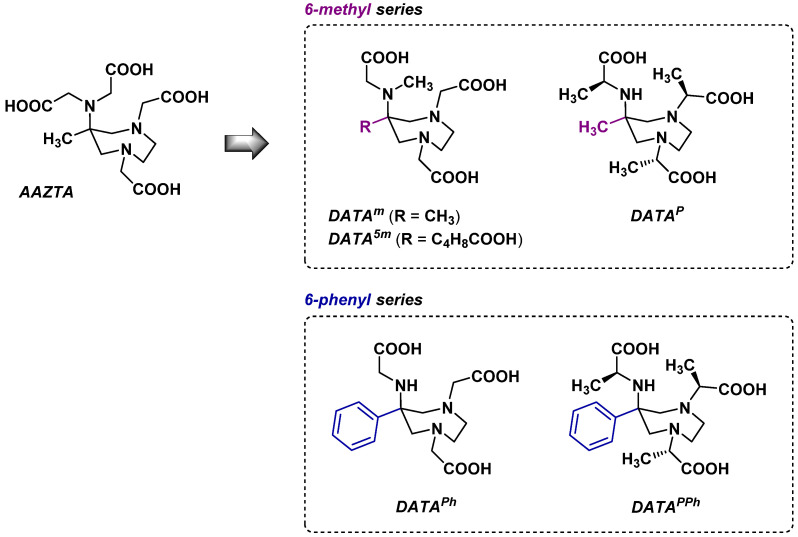
Structure of DATA chelators compared to AAZTA.

Dynamic NMR and 2D-NOESY experiments showed that both the 6-methyl and the 6-phenyl series bound ^68^Ga rapidly, the latter series forming one major stable solution species (pseudo-twisted-chair conformer [53]) at pH ≥ 3.5. Radiolabeling yields in both series were ≥96% within 3 min at RT over the pH range of 4 to 7 [54]. Gallium complexes in 6-methyl series displayed weaker in vitro stability profiles than their 6-phenyl analogues due to the formation of multiple species with different stability; however, no such instability was observed in vivo during preliminary biodistribution studies in Sprague Dawley rats. ^68^Ga-radiolabeling conditions of four DATA chelators (Figure 3) were deeper studied by the same team, highlighting their ideal characteristics for the development of PET imaging agent candidates [55]. The most promising results were obtained with DATA^m^, which reached the highest radiolabeling yields at the lowest chelator concentration (>97% in 1 min at 3.6 µM), although it seemed less permissive in terms of pH than the 6-phenyl derivatives (optimal labeling achieved at pH 5, with a noticeable decrease in the rates of complexation at pH 7). [^68^Ga]Ga-DATA complexes showed excellent stability in the presence of either apo-transferrin or DTPA (<3% free radioactivity after 120 min) and labeling kinetics comparable to NOTA. Interestingly, even though the higher degree of preorganization inducted by the bulkier substituent in the 6-phenyl series could have been a factor influencing the complexation parameters, the major difference between the two series was lipophilicity (log*P* [DATA^m^] = −4.02 vs. log*P* [DATA^Ph^] = −2.68). Thus, the choice between a 6-methyl and a 6-phenyl DATA chelator may influence the global hydrophilic/lipophilic balance of a vector candidate and have implications in its individual in vivo biodistribution.

**Scheme 9:**
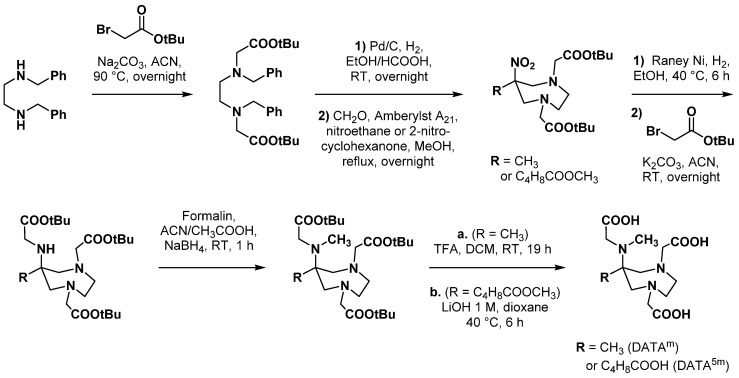
Five-steps synthesis of DATA^m^ and DATA^5m^ proposed by Farkas et al. [56].

Farkas et al. reported a slightly modified synthetic route to access DATA^m^ and its bifunctional analogue DATA^5m^, allowing the orthogonal protection of this latter compound for its use in bioconjugation reactions (Scheme 9) [56]. First, *N,N′*-dibenzylethylenediamine was *N*-dialkylated using *tert*-butyl bromoacetate. Debenzylation was catalyzed with Pd/C under hydrogen atmosphere; then, the reaction product was directly engaged in the Mannich condensation step, involving either nitroethane (DATA^m^) or 2-nitrocyclohexanone (DATA^5m^). Reduction of the nitro group with Raney nickel under hydrogen atmosphere formed the corresponding primary amine that could be mono-alkylated with *tert*-butyl bromoacetate and *N*-methylated by reductive amination. The expected chelating agent was finally obtained after deprotection with either TFA (DATA^m^) or lithium hydroxyde 1 M in dioxane (DATA^5m^).

The stability constants of [Ga-DATA^m^] and [Ga-DATA^5m^] complexes were slightly higher than [Ga-AAZTA] (log*K*_Ga-DATA(m)_ = 21.54 and log*K*_Ga-DATA(5m)_ = 21.41 vs. log*K*_Ga-AAZTA_ = 21.15) and comparable to [Ga-DOTA] [57]. However, the predominant species at physiological pH, [Ga-DATA^m^-OH]^−^ and [Ga-DATA^5m^-OH]^2−^, displayed quite lower stability than [Ga-AAZTA-OH]^2−^. Interestingly, the stability of complexes with DATA derivatives and several other metals, such as Ca, Mn [58], or Cu [59], are also lower than their AAZTA analogues. In near-physiological conditions, the [Ga-DATA^5m^] complex was characterized by a higher kinetic inertness than its DATA^m^ and AAZTA analogues (t_1/2_ = 44 h vs. 11 h and 21 h, respectively), with almost the same values in exchange reaction conditions with transferrin. The greater stability of [Ga-DATA^5m^] could be explained by the dissociation half-life of [Ga-DATA^5m^-OH]^2−^, which was four and two times higher than [Ga-DATA^m^-OH]^−^ and [Ga-AAZTA-OH]^2−^, respectively. Overall, the excellent complexation and kinetic properties of DATA^5m^ towards ^68^Ga have positioned it as a promising chelator and BCA in the conception of PET probes candidates. Currently, DATA^5m^ has been the only AAZ3A derivative that has progressed to clinical development.

## 3. Applications in Nuclear Medicine

Despite the undeniable kinetic improvements brought by DATA derivatives over AAZTA, this latter chelating agent was the most exemplified mesocyclic-complex-forming unit in innovative PET imaging probes. This could be explained by the more straightforward synthesis sequence of AAZTA, which is easier to deploy in proof-of-concept works. Several types of vectors, such as peptides, small molecules, or even monoclonal antibodies, have been used as substrates for bioconjugation with AAZTA-derived bifunctional agents.

### 3.1. Monoclonal Antibodies

**Scheme 10:**
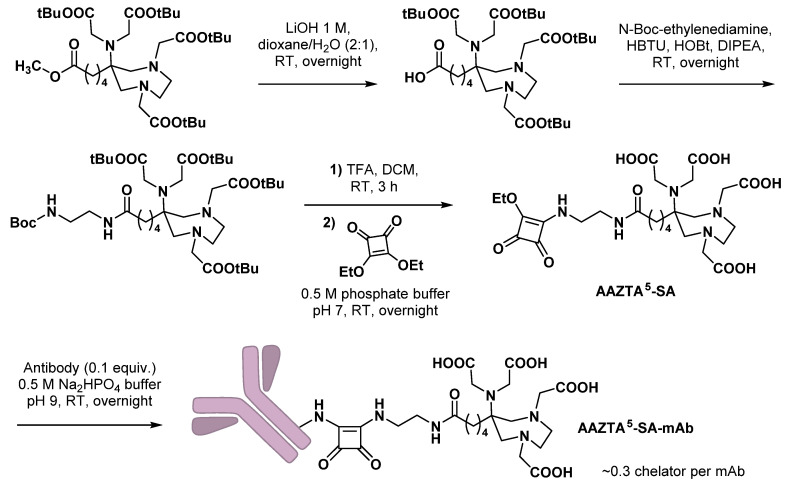
Synthesis of AAZTA^5^-SA bifunctional agent and bioconjugation reaction with a mAb, as reported by Klasen et al. [60].

Bifunctional chelating agents compatible with mild radiolabeling reaction conditions are particularly needed for monoclonal antibodies (mAbs) functionalization, as mAbs are heat- and pH-sensitive macromolecules. In this view, Klasen et al. combined an AAZTA^5^ scaffold with a squaramide ester motive and coupled this BCA to a model antibody (bevacizumab) [60]. This ligation technique, based on the pH-dependent reactivity of squaric acid diethyl ester, is increasingly used in the design of new radiopharmaceuticals [61]. Starting from protected AAZTA^5^, methyl ester could be selectively hydrolyzed with lithium hydroxide and the resulting carboxylic acid further coupled with *N*-Boc-ethylenediamine. After deprotection of both *tert*-butyl-ester- and Boc-protecting groups, the resulting compound was immediately coupled with squaric acid (SA) diethyl ester. Finally, bioconjugation with bevacizumab was conducted in mild conditions (Scheme 10). However, it led to a mean chelator-to-antibody ratio notably lower than the two DTPA-SA analogues used as references (0.29 ± 0.04 vs. 5.38 ± 2.69 and 6.06 ± 2.46). From a radioimmunotherapy perspective, AAZTA^5^-SA-mAb was radiolabeled with beta-emitting ^177^Lu to reach, after 90 min at pH 7 and RT, almost quantitative RCY. Optimization of this radiolabeling step highlighted the need to prior purify carefully the bioconjugate by steric exclusion chromatography to discard excess of unbound chelator. The [^177^Lu]Lu-AAZTA^5^-SA-mAb complex displayed high in vitro stability both in human serum and in PBS (>94% and >92%, respectively) within 15 days, as well as the two [^177^Lu]Lu-DTPA-SA-mAb references. Provided that [^177^Lu]-AAZTA^5^-SA-mAb remains stable in vivo, this study demonstrated the high potential of AAZTA-based bifunctional agents in the design of radioimmunoconjugates for therapeutic applications. Bioconjugation conditions should nevertheless be optimized to achieve a mean chelator-to-antibody ratio around four, considered optimal for this type of immunoconjugate.

### 3.2. Small Molecules

#### 3.2.1. Dual MRI/PET Imaging Agents

**Scheme 11:**
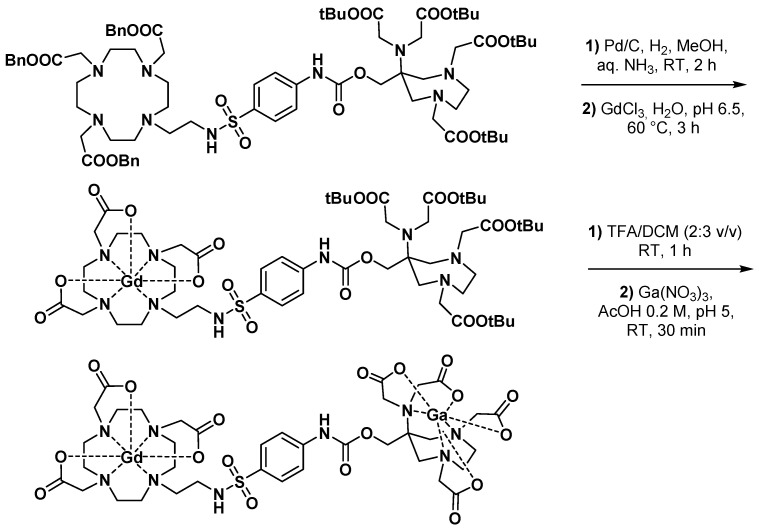
Synthesis and labeling of the DO3A-sulfonamide-AAZTA pH-sensitive described by Vologdin et al. [62].

One of the earliest PET imaging applications involving an AAZTA chelator was reported by Vologdin et al. and consisted of a heterodimeric DO3A-sulfonamide-AAZTA ligand presented as a potential pH-sensitive dual MRI/PET probe [62]. The pH-responsiveness of this molecule was due to the sulfonamide nitrogen that changed the hydration state of the Gd complexed with DO3A at pH > 8 [63], without interacting with the adjacent [^68^Ga]Ga-AAZTA complex. The main issue in the synthesis of this Gd-Ga heterobimetallic complex was the need for two different orthogonal protecting groups for the carboxylic acids of each chelator, DO3A and AAZTA, allowing sequential and selective complexations (Scheme 11). For this purpose, benzyl groups and *tert*-butyl esters were used to protect DO3A and AAZTA carboxylic acids, respectively. Although this heteroditopic complex effectively displayed in vitro the expected relaxivity variations over the pH range 4.0–9.3, it has not been evaluated in vivo in an attempt to quantify by PET a possible pH-dependent concentration of the probe.

#### 3.2.2. Bisphosphonate Derivatives

**Scheme 12:**
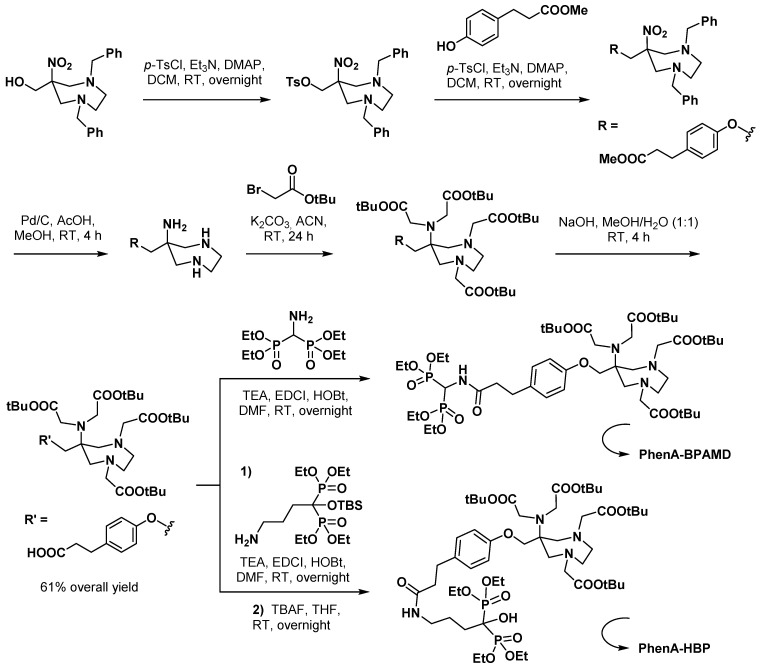
Synthesis of bisphosphonate-containing PET imaging agent candidates reported by Wu et al. [64].

Starting from AAZTA-MeOH, Wu et al. designed a phenoxy-containing BCA (PhenA) that was coupled to bisphosphonate derivatives [64]. These compounds were then radiolabeled with ^68^Ga and evaluated as bone PET imaging agents. PhenA could be obtained through a high-yield five-step synthesis (Scheme 12). After functionalization of the protected bisphosphonate, a final hydrolysis reaction allowed global deprotection of both the vector and the chelating part. Optimization of the ^68^Ga radiolabeling reaction conditions gave optimal RCY (93–98%) in 1 mL total volume, at pH 4.1 with 30 µM ligand (which is in accordance with vector amount in commercial kits [65,66]) and within 5 min at RT. These imaging agent candidates were suitable with easy-to-perform radiochemical purity (RCP) controls, either by HPLC or by TLC in a similar way to commercial kit-based ^99m^Tc-bisphosphonate radiopharmaceuticals. In vivo biodistribution study of [^68^Ga]Ga-PhenA-BPAMD showed comparable results to its DOTA analogue (11.0% vs. 9.21% dose/g bone uptake at 60 min); in contrast, the hydroxyl-containing derivative [^68^Ga]Ga-PhenA-HBP displayed a much lower bone uptake (only 3.8% dose/g at 60 min). Although these PET probes demonstrated a low liver uptake and a fast kidney excretion, their slow washout from the blood circulation suggested in vivo instability. Incubation of [^68^Ga]Ga-PhenA-BPAMD in human plasma at 37 °C supported this assumption, showing progressively decreasing RCP over time (82.3%, 68.8%, and 46.7% at 30, 60, and 120 min, respectively). This significant drawback will have to be solved to confirm the relevance of incorporating AAZTA in bone-targeted theranostic agents, bearing in mind that some DOTA-containing analogues are already evaluated in humans for ^68^Ga PET imaging [67] and ^177^Lu therapy [68,69].

#### 3.2.3. Curcumin Derivatives

**Scheme 13:**
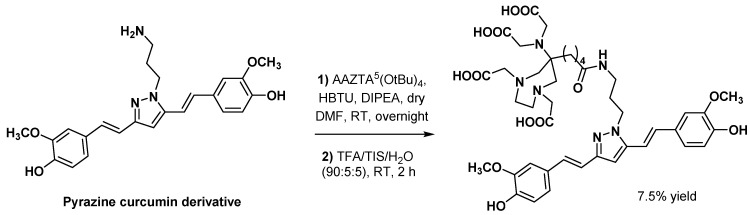
Conjugation of protected AAZTA^5^ chelator with a pyrazine curcumin derivative, as described by Orteca et al. [70].

To expand the potential applications of curcumin derivatives to nuclear medicine imaging of cancers, Orteca et al. developed new targeting vectors based on curcumin scaffolds functionalized with AAZTA or NODAGA chelators [70]. Synthesis of NODAGA-C21 and AAZTA-PC21 was performed in four and five steps, respectively, with pyrazole derivatization of the curcumin keto-enol moiety in the preparation sequence of AAZTA-PC21. A protected pentanoic acid AAZTA derivative (AAZTA^5^) was used as a bifonctionnal agent and was conveniently coupled with the pyrazine curcumin derivative via amide bond formation (Scheme 13). Both NODAGA-C21 and AAZTA-PC21 were radiolabeled with ^44^Sc and ^68^Ga, using a 10 nmol precursor in 0.25 M ammonium acetate pH 4 (^44^Sc labeling) or in 0.2 M sodium acetate pH 4 (^68^Ga labeling). Rapid kinetics were observed for the labeling of NODAGA-C21 with ^68^Ga (>95% incorporation yield after 1 min at 95 °C), while slow and poorly reproducible incorporation (<50% after 30 min at 95 °C) were experienced with ^44^Sc. Conversely, AAZTA-PC21 showed slightly slower kinetics but achieved RCY >95% after 10 min at 30 °C for both ^44^Sc and ^68^Ga, demonstrating the ability of AAZTA to complex these two radioelements under very mild conditions. Finally, although AAZTA-PC21 complexes displayed improved in vitro stability in human blood (around 60% after 2 h), this parameter still has to be studied in vivo to confirm the potential of these PET imaging candidates.

#### 3.2.4. Prostate-Specific Antigen Ligands

Based on the glutamate-urea-lysine (KuE) scaffold, prostate-specific membrane antigen (PSMA) ligands are small molecules of great interest in targeting prostate cancer cells, especially for theranostic applications [71]. To date, the two PSMA ligands reported being most frequently used for radioligand therapy are PSMA-617 and PSMA-I&T, both containing a DOTA chelator.

**Figure 4:**
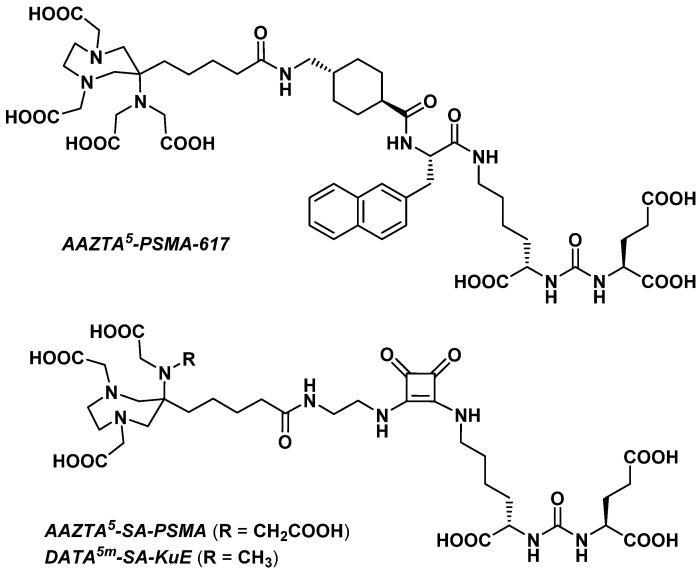
Structure of PSMA ligands functionalized with AAZTA-derived chelator.

Sinnes et al. explored the influence of the chelator part on the in vitro characteristics of a PSMA vector molecule by replacing DOTA with AAZTA^5^ in PSMA-617 [72]. The resulting AAZTA^5^-PSMA-617 (Figure 4) was successfully radiolabeled with ^68^Ga, ^44^Sc, and ^177^Lu at RT within 5 min, reaching >99% RCY for a chelator-to-radiometal molar ratio = 10:1. Both [^68^Ga]Ga- and [^44^Sc]Sc-AAZTA^5^-PSMA-617 were extremely stable in human serum, PBS, and EDTA/DTPA in PBS (>90% RCP over 2 h for the ^68^Ga derivative and >95% RCP over 8 h for the ^44^Sc derivative). In contrast, small degradations to 81% RCP were observed after 24 h for [^177^Lu]Lu-AAZTA^5^-PSMA-617 in human serum. Hence, beyond the type of chelator used, the nature of the vector agent (peptide or small molecule) seems to have a significant influence on this parameter. For all three complexes, in vitro binding affinity studies on PC3 cells yielded Ki values broadly comparable to corresponding DOTA analogues (8.7 to 30.6 nM vs. 4.7 to 6.9 nM, respectively). Cellular membrane to internalization ratios for each AAZTA^5^-PSMA-617 derivative were also in the same range as the radiolabeled DOTA tracers (13.0 to 20.0% IA/10^6^ cells vs. 15.8 to 17.7%IA/10^6^ cells, respectively). These favorable in vitro properties demonstrated the in vivo potential of AAZTA-containing PSMA ligands for theranostic applications.

The [^44^Sc]Sc-AAZTA^5^-PSMA-617 derivative was later evaluated in vivo after an in-depth study of the radiolabeling reaction conditions [73]. Although its amount in radiopharmaceuticals is strictly limited by European Pharmacopoeia [74], HEPES 0.1 M was found to be a particularly suitable buffer to reach high RCY and excellent molar activity (average of 39.4 GBq/µmol) at pH 4. To achieve quantitative RCY at neutral pH, a higher peptide concentration (10 µM rather than 1 µM) was necessary. [^44^Sc]Sc-AAZTA^5^-PSMA-617 confirmed its stability in human plasma over 24 h, and in vitro cellular uptake studies showed a higher accumulation of AAZTA-containing radioligands in LNCaP cells compared to their DOTA-containing analogues. Preclinical PET imaging and ex vivo biodistribution studies with [^68^Ga]Ga- and [^44^Sc]Sc-AAZTA^5^-PSMA-617 in LNCaP tumor-bearing mice concluded a higher accumulation of the [^44^Sc]Sc-AAZTA derivative in tumors than its DOTA analogue (14.98 vs. 7.49 %ID/g, 3 h post-injection), possibly due to a two times higher molar activity. Self-blocking experiments confirmed the PSMA specificity of these radioligands. Overall, the good radiolabeling characteristics and intrinsic physicochemical properties of [^44^Sc]Sc-AAZTA^5^-PSMA-617 allowed its good in vivo imaging performance on a xenografted mouse model versus one of the most relevant PSMA derivatives used in clinics, i.e., DOTA-PSMA-617, confirming the potential of this AAZTA-conjugate for clinical translation. Further studies comparing the in vivo behavior of AAZTA^5^- and DOTA-PSMA-617 radiolabeled with ^68^Ga and ^177^Lu would adequately complete this work.

Alongside these encouraging results, the same team developed a functionalization procedure of KuE based on squaric acid reactivity [75]. The resulting AAZTA^5^-SA-PSMA was obtained after a single coupling step in 23% yield and radiolabeled with either ^68^Ga or ^44^Sc in excellent yields (>95% RCY), in usual reaction conditions (0.2 M ammonium acetate pH 4.5, RT, 3 min, and 0.25 M sodium acetate pH 4, RT, 3 min, respectively). The optimal amount of vector was determined to be 10 nmol; for ^44^Sc radiolabeling, it is noteworthy that RCYs were slightly lower for a larger amount of vector. AAZTA^5^-SA-PSMA was also radiolabeled with ^64^Cu (10 nmol vector, 0.2 M acetate buffer pH 5.5, RT) and ^177^Lu (10 nmol vector, 0.25 M HEPES pH 4.4, RT) in excellent RCY (>95%) within only 1 min. Both ^68^Ga and ^44^Sc were stable in human serum; ^177^Lu complex showed a slightly decreased stability after 48h (86% RCP), while the ^64^Cu-labeled derivative was poorly stable after 12 h (40% RCP). This work highlighted the highly versatile labeling properties of AAZTA^5^-SA-PSMA for several radiometals, suggesting the significant value of the AAZTA-SA building block in the design of new radiopharmaceuticals candidates. A DATA^5m^ analogue of this latter molecule, named DATA^5m^-SA-KuE, was also synthesized and radiolabeled with ^68^Ga [76]. Interestingly, reaction heating was necessary to reach high RCYs, especially when small amounts of vector were used. In vivo biodistribution study of [^68^Ga]Ga-DATA^5m^-SA-KuE compared to [^177^Lu]Lu- and [^44^Sc]Sc-AAZTA^5^-SA-PSMA evidenced no impact of the radioelement on the pharmacokinetic properties of the radioconjugates. In µPET imaging, squaric acid-conjugates showed similar tumor accumulation and lower kidney uptake than ^68^Ga-PSMA-11. In this work, the radiolabeling of both AAZTA^5^-SA-PSMA and DATA^5m^-SA-KuE with ^68^Ga, ^177^Lu, and ^44^Sc would have been of great interest to compare the potential of either chelator in the design of vectors compatible with theranostic pairs. It can be assumed, however, that the N3O3 donor set of DATA^5m^ would be far from optimal to complex Lu^3+^, which usually requires hepta- or octavalent chelators [77]. The most appropriate theranostic pair to fit AAZTA-derived chelators would certainly be ^44^Sc/^47^Sc, although this has not been formally reported in the literature.

#### 3.2.5. Fibroblast Activation Protein Inhibitors

**Scheme 14:**
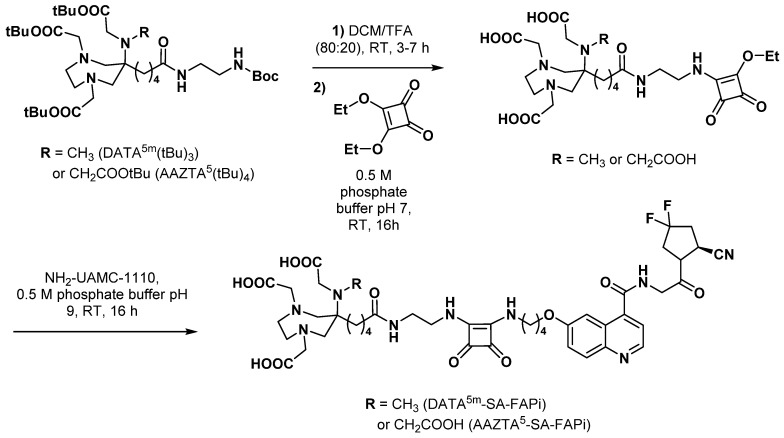
Synthesis of AAZTA^5^- and DATA^5m^-SA-FAPi derivatives [82,84].

Fibroblast activation protein α (FAP) is an endopeptidase that is found upregulated in various tumor types. It is an important marker of cancer-associated fibroblasts that appears to contribute to some of their tumor-promoting activities [78]. After the identification of the FAP inhibitor (FAPi) lead molecule UAMC-1110 [79], many FAP-targeting imaging tools based on the same quinoline scaffold were designed for theranostic applications in a variety of cancers [80,81]. For that purpose, the use of the squaramide motif to anchor a chelating moiety on a vector molecule has been exemplified with FAPi compounds: the team of Frank Rösch synthesized and evaluated a DATA^5m^-SA-FAPi derivative in comparison with its DOTA analogue [82]. Starting from the quinoline NH_2_-UAMC-1110, both DOTA and DATA^5m^-bearing molecules were obtained following a convenient preparation procedure that relied on the simple coupling conditions allowed by the pH-dependent reactivity of SA (Scheme 14). DOTA- and DATA^5m^-SA-FAPIs retained their affinity for FAP (low nanomolar IC_50_ values), either uncomplexed or complexed with *^nat^*Ga (DOTA and DATA^5m^ derivatives) and *^nat^*Lu (DOTA derivative only). As expected, comparison of the radiolabeling reaction conditions of the two derivatives showed that ^68^Ga complexation with DATA^5m^ was less demanding than DOTA (RCY >98% within 1 min at RT in 300 µL 1 M ammonium acetate pH 5.5 with 16 nmol precursor, vs. RCY >97% within 5 min at 95 °C with the same amount of buffer and precursor). For each chelator, a minimal precursor amount of >15 nmol was identified as a key parameter to reach quantitative yields. [^68^Ga]Ga-DATA^5m^-SA-FAPi remained stable (RCP >95%) in vitro in PBS, NaCl 0.9%, and human serum over 2 h. In vivo evaluation of [^68^Ga]Ga-DOTA-SA-FAPi in HT-29 tumor-bearing mice showed clear tumor accumulation (SUV_mean_ = 0.75 ± 0.09 and overall tumor uptake = 5.2 %ID/g 60 min post-injection) with low uptake in most of the other organs. A comparative in vivo study of the two SA-FAPi would have been of great interest to assess the interest of DATA^5m^ over reference chelator DOTA. Nevertheless, successful clinical use of [^68^Ga]Ga-DATA^5m^-SA-FAPi was later reported and highlighted the possible interest of this radiotracer in benign liver tumor imaging [83]. Interestingly, the radiolabeling reaction was scaled-up for human use and was carried out in 3.34 ± 0.04 mL 0.08 M ammonium acetate pH 3.6 heated at 50 °C for 8 min (RCY > 92%).

The same team also synthesized and studied the AAZTA^5^ analogue of DATA^5m^-SA-FAPi, following a strictly comparable synthesis sequence (Scheme 14) [84]. The broader coordination capabilities of AAZTA allowed ^68^Ga, ^44^Sc, and ^177^Lu radiolabeling with direct comparison with DOTA-SA-FAPi. Excellent complexation properties of AAZTA over DOTA were especially evidenced during ^44^Sc labeling experiments: quantitative labeling was reached for ≥5 nmol precursor within 5 min with AAZTA^5^-SA-FAPi vs. 40 nmol precursor within 15 min with DOTA-SA-FAPi. Gallium-68 and scandium-44 AAZTA^5^-SA-FAPi complexes were very stable in human serum (at least >97% RCP over 2 h and 8 h, respectively); however, the ^177^Lu derivative showed increasing instability over time, although RCP remained >96% after 6 h. As expected, with the presence of four carboxyl groups and ionic bonds between chelator and radiometal, AAZTA complexes were highly hydrophilic (logD_7_._4_ = −2.53 and −2.50 for ^68^Ga and ^44^Sc derivatives, respectively), positioning them as promising radiopharmaceuticals candidates for theranostic use, provided conclusive in vivo results are met.

### 3.3. Peptides

Due to their reasonable convenience of synthesis, adapted pharmacokinetic behavior, and high target specificity, peptide probes are widely exploited in the conception of theranostic agents in nuclear medicine. The first radiolabeled peptide used in humans was the somatostatin analogue [^123^I]I-204-090 developed in 1989 [85], which paved the way to [^111^In]In-DTPA-Octreotide [86] and several other DOTA-containing somatostatin analogues [87,88,89]. In addition to the search for various new targets, novel complexing agents such as AAZTA are being evaluated to allow easy and rapid radiolabeling of these peptide vectors with radiometals.

#### 3.3.1. RGD Peptides

**Scheme 15:**
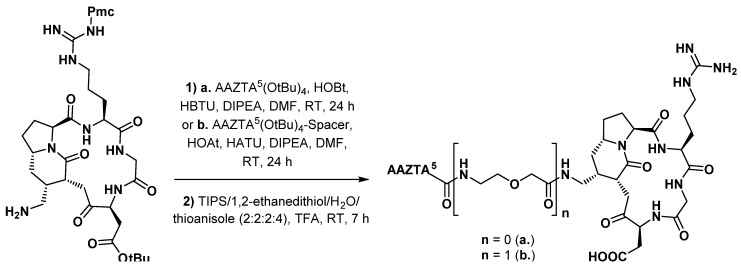
Functionalization of the RGD peptidomimetic DB58 with AAZTA chelating agents, as proposed by Manzoni et al. [90].

Functionalization of a peptide with AAZTA^5^ was first described by Manzoni et al. [90], who prepared about ten RGD peptidomimetic conjugates bearing different chelating groups. Modified integrin α_V_β_3_ ligand DB58, incorporating an azide group, was chosen for functionalization. After hydrogenation with Pd/C to get the corresponding amine, it was coupled to either AAZTA^5^(OtBu)_4_ or its spacer-containing analogue, using standard amide bond formation procedures (Scheme 15). AAZTA^5^-DB58 was radiolabeled with ^68^Ga on an automated synthesis module (Taddeo^®^, Comecer) in sodium acetate buffer pH 4.6 incubated at RT for 10 min. An average RCP of 93% and specific activity of 26–29 MBq/nmol were reached without further purification. Evaluation on a U-87 MG xenograft mouse model of glioblastoma showed a highlighted and readily detectable tumor 40 min post-injection. The expected competitive antagonism was observed by simultaneous injection of the integrin ligand *c*(RGDfV). Furthermore, the length of the spacer proved to be a crucial parameter for the binding affinity of the radioconjugate. This early work suggested the in vivo potential of AAZTA derivatives in the development of new peptide-based PET probes.

In 2017, Nagy et al. also developed an AAZTA-RGD derivative radiolabeled with ^44^Sc, which was studied in 4T1 tumor-bearing BALB/c mice [17]. High radiotracer accumulation in the tumor (25-fold higher than the background) could be observed at 90 min post-intravenous injection, the long half-life of ^44^Sc providing an opportunity for delayed imaging so that the radiotracer could clear from non-targeted background organs.

#### 3.3.2. Gastrin Analogues

**Figure 5:**
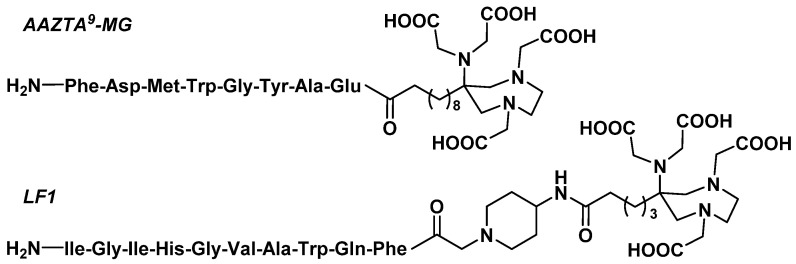
Structure of AAZTA^9^-MG and LF1.

Cholecystokinin 2 receptor (CCK2r), which is overexpressed in various tumor tissues (e.g., medullary thyroid carcinoma [91] or gastrointestinal stromal tumors [92]), can be targeted with gastrin analogues [93]. In this light, Pfister et al. designed an AAZTA-containing gastrin (10–17) (minigastrin, MG) analogue as an original theranostic tool targeting CCK2r (Figure 5) [94]. The AAZTA^9^(OtBu)_4_ bifunctional agent was coupled after HATU/HOAt activation on the N-terminal extremity of the peptide at the end of its synthesis on Rink Amide resin. A mixture of TFA/TIPS/H_2_O (95:2.5:2.5) allowed both cleavage of the peptide from the resin and its deprotection. Radiolabeling assays of AAZTA^9^-MG showed >95% RCY after 10 min at RT using appropriate pH 4.5 buffered solutions for ^68^Ga (1.14 M sodium acetate trihydrate), ^177^Lu, and ^111^In (0.3 mM ascorbic acid). These mild reaction conditions are particularly suitable for minigastrin, containing a methionine sensitive to oxidation. These radioconjugates showed a high in vitro hydrophilicity (logD = −3.6 for [^68^Ga]Ga-AAZTA^9^-MG and logD = −3.73 for [^177^Lu]Lu-AAZTA^9^-MG) and noticeable in vitro instability, either in human serum for [^68^Ga]Ga-AAZTA^9^-MG (81 ± 7.9% RCP after 2 h) or in DTPA solution for [^177^Lu]Lu-AAZTA^9^-MG (77 ± 0.72% RCP after 2 h). In vivo biodistribution study in BALB/c mice confirmed the limited stability of the ^68^Ga compound (high blood levels, high plasma protein binding) and evidenced partial hepatobiliary excretion of ^177^Lu and ^111^In derivatives, which could be suboptimal for theranostic approaches. Despite its good CCK2r+ tumor-targeting properties, [^68^Ga]Ga-AAZTA^9^-MG instability resulted in a tumor-to-blood ratio of about 4 with 1.5 %ID/g in the tumor compared to 0.4%ID/g in blood. As higher contrast was reported for [^68^Ga]Ga-DOTA-MG [95], pharmacokinetic limitations of AAZTA-containing MG derivatives required further optimization efforts.

Gastrin releasing peptide receptor (GRPr) is another G protein-coupled receptor that was successfully exploited for peptide-receptor-targeted theranostic applications in oncology [96]. Among the radiotracers targeting GRPr, analogues of the octapeptide bombesin (7–14) were extensively studied, including in patients [97]. Starting from a GRPr peptide antagonist, Hofstetter et al. functionalized the *N*-terminal part of this peptide with a positively charged 4-amino-1-carboxymethylpiperidine spacer and an AAZTA^5^ BCA (Figure 5) [98]. The synthesis method was strictly comparable to that of AAZTA^9^-MG. The peptide conjugate, later called LF1, was radiolabeled with ^68^Ga on an automated synthesis module (Modular-Lab PharmTracer^®^, Eckert & Ziegler, Berlin, Germany) in 0.2 M sodium acetate buffer pH 4 at RT within 5 min. The ^68^Ga eluate was purified on a cation exchange cartridge, and the final radioconjugate underwent SepPak C-18 purification to reach around 90% RCP. LF1 was also labeled with ^177^Lu and ^111^In in 98% RCY in 0.5 M ammonium acetate buffer pH 5.4 without further purification. As well as AAZTA^9^-MG derivatives, LF1 radioconjugates showed a hydrophilic profile (LogD ranging from −2.8 to −3.2). Saturation binding studies confirmed that functionalization did not compromise the affinity of the vector for its target receptor for ^177^Lu and ^111^In derivatives. Conversely, [^68^Ga]Ga-LF1 affinity for GRPr was three times lower than [^177^Lu]Lu-LF1. In vitro internalization studies also evidenced that total cell-associated uptake and percentage of the specific internalization fraction were considerably lower for [^68^Ga]Ga-LF1, hampering its applicability as a suitable in vivo PET tracer candidate.

#### 3.3.3. Octreotide Analogues

Somatostatin receptor (sstr) ligand analogues are the most well-developed and extensively investigated group of small peptides functionalized by a chelating agent [99]. Since the regulatory approval of [^111^In]In-DTPA^0^-octreotide for scintigraphic imaging of sstr expression [100], modulations were conducted both on the peptide and on the chelator part of these imaging agents to optimize their selectivity, receptor binding affinity, internalization, pharmacokinetic properties, or radiolabeling abilities.

**Scheme 16:**
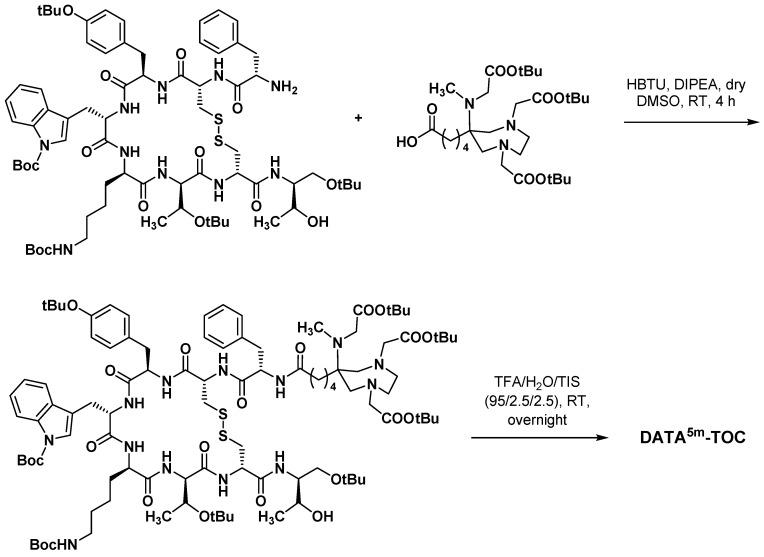
Synthesis of DATA^5m^-TOC according to Seeman et al. [102].

To evaluate the properties of a mesocyclic ligand on this type of targeting vector, AAZTA^5^-TOC was synthesized via a simple peptide coupling between AAZTA^5^ and Phe^1^-Tyr^3^-octreotide (TOC) [101]. Radiolabeling with ^68^Ga, ^44^Sc, and ^177^Lu reached high RCY after 1 min reaction time at RT with optimized precursor amounts (10 nmol for ^68^Ga and ^44^Sc assays; 1:10 lutetium-to-precursor molar ratio for ^177^Lu assay). Interestingly, pH variations in the 4.5–5.5 range had no influence on labeling speed or yields. Concerning in vitro stability, only [^68^Ga]Ga-AAZTA^5^-TOC showed slight instability (85% RCP) and sensitivity to transchelation (40–50% RCP) in human serum after 2 h. Despite the promising preliminary results of AAZTA^5^-TOC, its development has not yet reached clinics. In contrast, its analogue, DATA^5m^-TOC, described 2 years before, attained the clinical development stage. Initial work by Seeman et al. on this PET tracer candidate consisted of elaborating a kit-type labeling protocol at RT [102]. A two-step sequence very similar to AAZTA^5^-TOC afforded DATA^5m^-TOC in 31% yield (Scheme 16). Then, ^68^Ga radiolabeling assays were carried out using four different eluate post-processing methods. Remarkably, >95% RCYs were achieved within 1 min at pH 4.9 and RT with fractionated and acetone post-processing. Peptide concentration, slightly higher in these two conditions, may also have an influence on these results. Regardless of the post-processing method, >98% RCYs were reached within 10 min in each case. Noteworthy, these experiments were conducted with only 100 MBq ^68^Ga and under agitation (400 rpm), which does not exactly match with routine radiolabeling conditions. Furthermore, even if labeling with acetone post-processed ^68^Ga confirmed its excellent properties for the preparation of high RCP radioconjugates [103], further purification steps would be required at the end of the synthesis as acetone is not approved for in vivo applications.

In the continuity of this pharmacotechnical development, Nock et al. proposed a direct comparison study of gallium-labeled DATA^5m^-TOC and DOTATOC, both in vitro and in xenograft mouse models [104]. After successful labeling with either ^nat^Ga or ^67^Ga (in view of the longer half-life of this gamma-emitting isotope compared to ^68^Ga: 3.3 days vs. 67.7 min), binding affinity assays showed that both ^nat^Ga-DATA^5m^-TOC and ^nat^Ga-DOTATOC were human somatostatin receptor type two (hsstr_2_)-preferring. Both conjugates underwent cell internalization process in AR42J and HEK293-hsstr_x_ cell lines, with significantly slower kinetics for ^nat^Ga-DATA^5m^-TOC. In line with these results, biodistribution study in AR42J tumor-bearing mice showed higher tumor uptake for [^67^Ga]Ga-DOTATOC than [^67^Ga]Ga-DATA^5m^-TOC (37.10 ± 10.37 %ID/g vs. 22.31 ± 1.87 %ID/g). Of particular interest is the fact that [^67^Ga]Ga-DATA^5m^-TOC showed lower accumulation and faster clearance from most physiological tissues than [^67^Ga]Ga-DOTATOC, resulting in higher tumor-to-background ratios. A biodistribution study in HER293-hsstr_2_ tumor-bearing mice gave comparable results.

**Figure 6:**
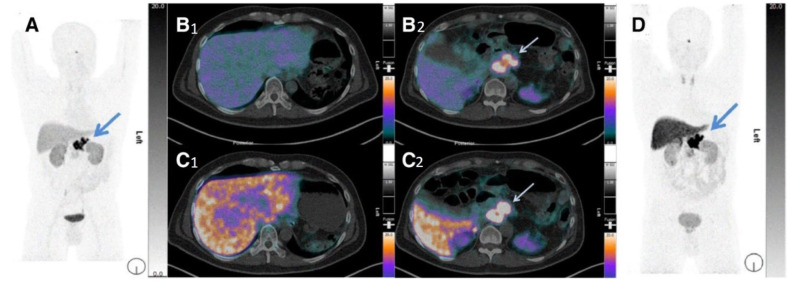
Comparative PET/CT images of [^68^Ga]Ga-DATA^5m^-TOC and [^68^Ga]Ga-DOTATOC as published by Sinnes et al. [105]. [^68^Ga]Ga-DATA^5m^-TOC PET MIP (**A**) and transverse PET/CT fusion images (**B_1_**,**B_2_**) show significantly lower uptake in normal liver compared with the same images with [^68^Ga]Ga-DOTATOC ((**D**) and (**C_1_**,**C_2_**), respectively). Blue arrows indicate high uptake in the primary pancreatic NET.

DATA^5m^-TOC single vial cold kit formulation was then transferred to the clinic, with a direct comparison between [^68^Ga]Ga-DATA^5m^-TOC and [^68^Ga]Ga-DOTATOC in a 46-year-old male patient with well-differentiated pancreatic NET lesions and peritumoral lymph nodes metastases [105,106]. Imaging was performed before the second cycle of 5 GBq [^90^Y]Y-DOTATOC. Compared with pretreatment imaging, post-treatment [^68^Ga]Ga-DOTATOC demonstrated partial remission with a 65% decrease in the uptake in the primary pancreatic tumor. [^68^Ga]Ga-DATA^5m^-TOC imaging performed 24 h later at the same time post-injection (50 min) showed a similar and very intense uptake in the primary pancreatic tumor. More importantly, notably lower uptake of [^68^Ga]Ga-DATA^5m^-TOC in the normal liver was observed compared to [^68^Ga]Ga-DOTATOC (SUV_max_ = 9.1 vs. 23.1; tumor-to-liver ratio = 5.2 vs. 3.1) (Figure 6). Overall, this first-in-human evaluation showed that the chelator switch from DOTA to DATA^5m^ did not compromise the biological properties of the peptide vector and allowed practical advantages of DATA for single-vial cold-kit formulation.

**Figure 7:**
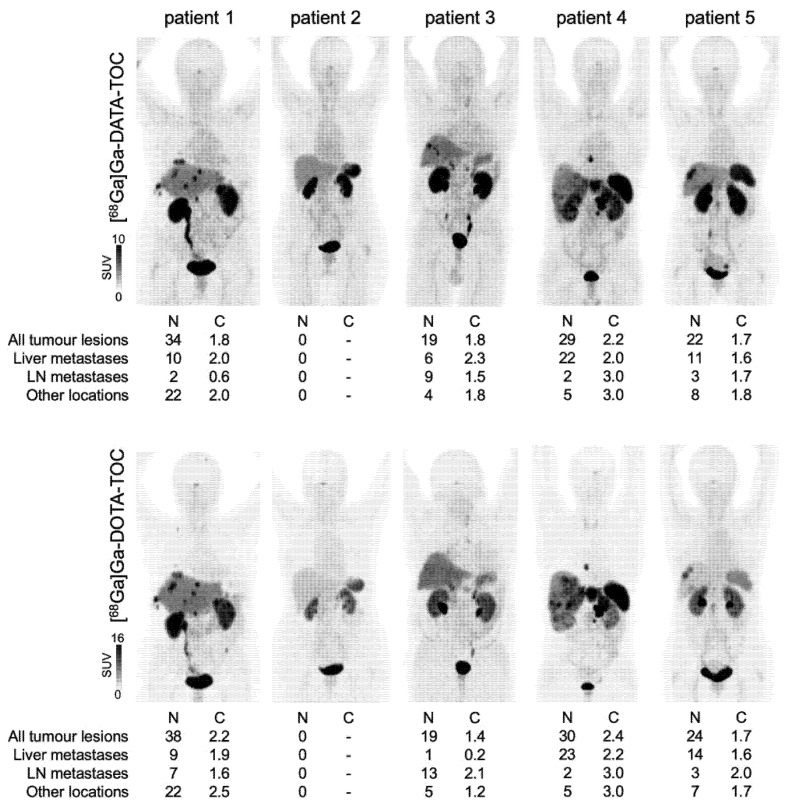
Comparative PET MIP images of [^68^Ga]Ga-DATA^5m^-TOC (top row) and [^68^Ga]Ga-DOTATOC (bottom row) in 5 patients, as published by Gaertner et al. [107]. No significant difference in lesion detectability was evidenced between the 2 radiotracers.

This proof of concept paved the way for a larger study in which a group of 19 patients who underwent [^68^Ga]Ga-DATA^5m^-TOC PET/CT was retrospectively compared to a group of 19 clinically stable patients who underwent [^68^Ga]Ga-DOTATOC PET/CT [107]. Both radiotracers were synthesized following a GMP-compliant procedure, using an automated cassette module (GAIA^®^, Elysia-Raytest) with ~1 GBq ^68^Ga (processed on SCX cartridge) and 40 to 50 µg peptide. [^68^Ga]Ga-DATA^5m^-TOC was obtained with 89.93 ± 16.9% RCY after C18 purification. Volume activity and apparent molar activity reached 78.00 ± 21.61 MBq/mL and 24.70 ± 5.95 MBq/nmol, respectively, with >95% RCP. In patients, uptake of [^68^Ga]Ga-DATA^5m^-TOC in the adrenal gland, pituitary gland, liver, and spleen was significantly lower compared to [^68^Ga]Ga-DOTATOC. This was also the case in primary tumor lesions and liver and lymph nodes metastases. In contrast, [^68^Ga]Ga-DATA^5m^-TOC uptake was higher in the blood pool and in the lungs. Although a trend for a higher detectability and conspicuity of liver metastases was observed for [^68^Ga]Ga-DATA^5m^-TOC (probably due to the lower physiological background uptake in this tissue), no significant differences were observed between this new tracer and [^68^Ga]Ga-DOTATOC regarding lesion detectability in this small number of patients (Figure 7). This apparent non-inferiority of [^68^Ga]Ga-DATA^5m^-TOC versus its DOTA analogue is an essential consideration in favor of its clinical relevance.

Finally, the diagnostic efficacy of [^68^Ga]Ga-DATA^5m^-TOC was compared with [^68^Ga]Ga-DOTA-NOC in 50 patients with gastroenteropancreatic (GEP) NET who underwent PET/CT imaging with both radiopeptides within a period of 1 to 10 days [108]. Both radiotracers were synthesized manually, with pre-concentration of the ^68^Ga eluate on an SCX cartridge eluted with 97.7% acetone/0.05 M HCl in the reaction vial containing 30 µg peptide and 1 mL of 0.4 M sodium acetate pH 4 buffer. DATA^5m^-TOC was radiolabeled at RT for 10 min and purified on a C18 cartridge, yielding 625 ± 266 MBq radiopeptide bulk solution prepared in 15 min (vs. 30 min for [^68^Ga]Ga-DOTA-NOC). In patients, biodistribution and image quality of both radiotracers were similar on visual examination. Slightly lower physiological liver uptake was confirmed for [^68^Ga]Ga-DATA^5m^-TOC over [^68^Ga]Ga-DOTA-NOC (SUV_max_ = 7.65 ± 5.37 vs. 8.94 ± 5.95). On a patient-wise analysis, both radiotracers were lesion-positive in 44 patients and negative in 6 patients. On a lesion basis, [^68^Ga]Ga-DATA^5m^-TOC had 98.6% concordance with [^68^Ga]Ga-DOTA-NOC (232 out of 235 lesions detected). Target tumor SUV_max_ and target-to-liver SUV_max_ ratios were not significantly different between the two derivatives, confirming the effective pharmacological and clinical imaging profile of [^68^Ga]Ga-DATA^5m^-TOC compared to its DOTA-containing analogues.

## 4. Conclusions

Since its early patenting [109], AAZTA has positioned itself as a promising chelating agent for imaging applications, opening the way to a family of compounds halfway between the well-known linear and macrocyclic chelating agents. The design and fairly convenient synthesis of various analogues of AAZTA allowed us to investigate their kinetic and thermodynamic properties with several metal ions. However, only AAZTA and DATA^5m^ were used in the development of original vector molecules for nuclear medicine applications. Their reactivity at room temperature and their compatibility with a wide pH range may pave the way for the design of theranostic agents based on pH- or heat-sensitive biomolecules. Regarding the conception and evaluation of new AAZTA-containing probes, the works described in this review highlighted the need for a systematic validation approach, with careful in vitro measurement of physicochemical parameters, stability, and affinity of the AZZTA-conjugate with its target. These results should be confirmed with in vivo biodistribution studies and microPET imaging on relevant animal models, possibly with several radioelements in a theranostic perspective. Only a few AAZTA-containing molecules have yet been evaluated in patients for imaging applications. It is reasonable to think that the therapeutic applications of these same vectors will take even longer to be evaluated in humans, considering the complexity for therapeutic radiopharmaceuticals to reach clinical evaluation stages. During the radiolabeling step, a significant portion of the final RCY can be achieved within minutes, making AAZTA particularly suitable for short half-life radioelements such as ^68^Ga. With shorter preparation times, higher batch activities, and possible cost-effective single vial cold kit formulation, the use of AAZTA-derived chelators could facilitate the clinical transfer of vector candidates and encourage even more the expansion of theranostic approaches in nuclear medicine.

## Data Availability

Data is contained within the article.
